# Supplementary material for: Vascular Calcification Is Associated with Fetuin-A and Cortical Bone Porosity in Stone Formers
Source: J Pers Med. 2022 Jul 10;12(7):1120. doi: 10.3390/jpm12071120 (PMC9319706; doi:10.3390/jpm12071120)
Supplement: Supplementary file 1 [file jpm-12-01120-s001.zip › jpm-1778213-supplementary.pdf]

Supplementary Table S1. 24-hour lithogenic urinary parameters of SF divided according to their mean AAC

	**Total** **n= 62**	**G1** **AAC <5.8%** **n= 33**	**G2** **AAC ≥5.8%** **n= 29**	**P value**
Volume, ml/24h	1800 (1440 - 2320)	1660 (1390 - 2260)	1600 (1350 - 2150)	0.66
Calcium, mg/24h	230.5 (147 – 301)	256 (143 - 300)	221 (140 - 289)	0.92
Sodium, mEq/24h	211.3 ± 84.6	193.2 ± 83.2	231.9 ± 82.9	0.07
Phosphate, mg/24h	859.1 ± 273.8	796.0 ± 245.9	928.9 ± 290.2	0.06
Uric acid, mg/24h	633.0 (475.0 – 788.0)	593.5 (437.3 – 743.8)	685.0 (530.5 – 781.5)	0.23
Oxalate, mg/24h	23.7 ± 8.8	25.8 ± 8.6	20.5 ± 8.5	0.01
Magnesium, mg/24h	87.0 (67.0 – 109.0)	78.5 (63.0 – 97.5)	86.0 (67.0 – 109.0)	0.22
Potassium, mEq/24h	55.0 (40.0 – 69.0)	48.0 (37.0 – 58.0)	58.0 (42.3 – 66.8)	0.10
Citrate, mg/24h	327.5 (238.5 – 403.1)	309.7 (206.5 – 448.5)	344.0 (271.0 – 434.6)	0.84
Urea, g/24h	13.6 ± 5.2	12.8 ± 6.1	15.1 ± 5.4	0.15
pH	6.1 ± 0.3	6.1 ± 0.6	6.0 ± 0.5	0.70

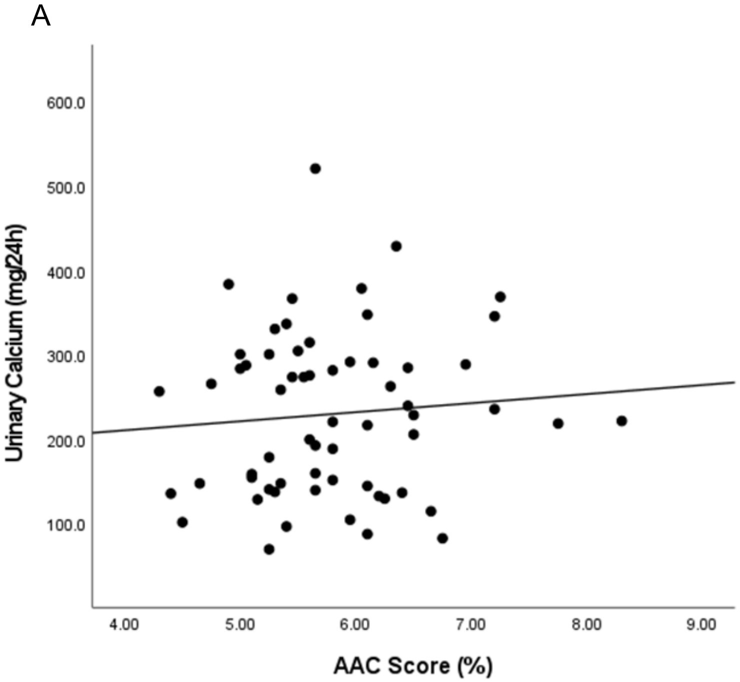

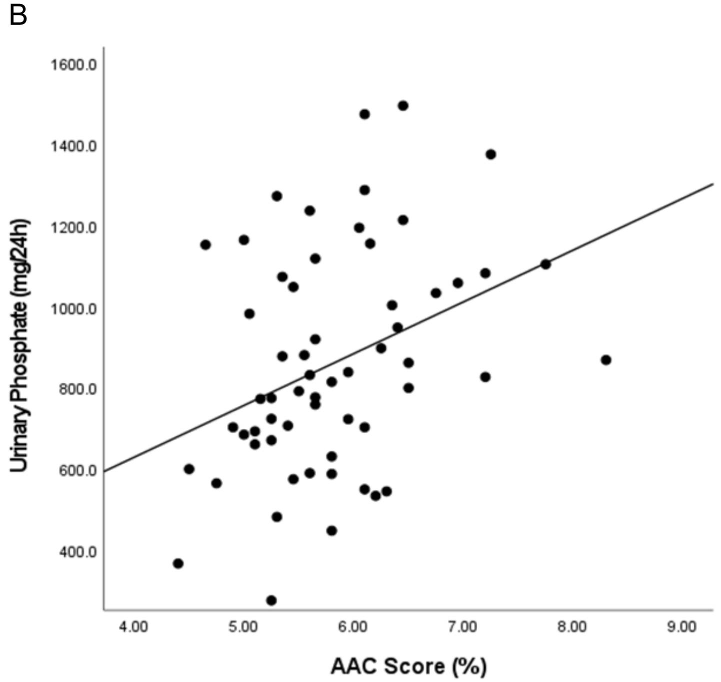

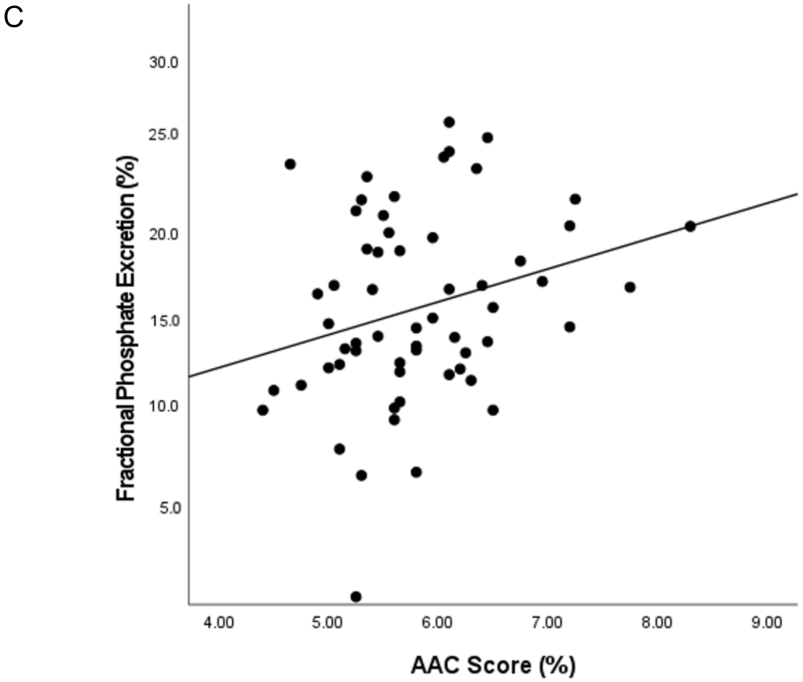

Supplementary Figure S1. Scatterplots between AAC Score and lithogenic urinary parameters in stone formers (SF). A - Urinary calcium: r = 0.05 (p=0.64); B – Urinary Phosphate: r= 0.37 (p=0.03); C – Fractional phosphate excretion r=0.27 (p=0.04).
